# Breakthrough mother-to-child transmission of HIV in a low-health facility in Uganda

**DOI:** 10.1016/j.ijregi.2023.07.009

**Published:** 2023-08-09

**Authors:** Benjamin Okello, Harriet Nyana, Richards Luwukya, Moses Odongkara, Winnie Kibone, Felix Bongomin

**Affiliations:** Department of Medical Microbiology and Immunology, Faculty of Medicine, Gulu University, Gulu, Uganda

**Keywords:** Mother-to-child transmission of HIV, Nevirapine, Breakthrough infection

## Abstract

**Objectives:**

Mother-to-child transmission (MTCT) of HIV can be minimized using elimination of MTCT (eMTCT) services. This study aimed to determine the proportion of infants who tested positive for HIV despite receiving eMTCT services in a rural setting in Northern Uganda.

**Methods:**

We retrospectively reviewed the early infant diagnosis register for the year January 2019 through June 2021 to collect data on participants enrolled in eMTCT services at Lalogi Health Centre IV in Omoro district, Uganda. Breakthrough HIV infection was defined as a positive HIV RNA on a dried blood sample at 18 months in a patient who received eMTCT services as recommended by national guidelines.

**Results:**

A total of 118 infants were enrolled in the study, 64 (54.2%) of whom were female. Most of the participants (n = 111, 94.1%) were on nevirapine prophylaxis for at least 6 weeks, 115 (97.5%) were exclusively breastfed, two (1.7%) were on complementary feeding, and one (0.8%) was not breastfed. Only five (4.2%) infants were lost to follow-up, four (3.4%) had incomplete data, and three (2.5%) had breakthrough HIV infections (positive HIV RNA and HIV antibody tests). All three cases of breakthrough HIV infection (one male and two female infants) were born to mothers who were diagnosed with HIV at delivery and were on nevirapine prophylaxis for less than 6 weeks.

**Conclusion:**

Our findings indicate that while eMTCT services were largely successful in minimizing vertical transmission of HIV in the rural setting in Northern Uganda, there were still some cases of breakthrough HIV infection associated with non-adherence to nevirapine prophylaxis and delayed maternal HIV diagnosis. Therefore, adhering to the national guidelines on nevirapine prophylaxis for at least 6 weeks for children born to mothers with HIV is recommended to further reduce the risk of vertical transmission of HIV.

## Highlights

• Nevirapine prophylaxis minimizes mother-to-child transmission of HIV.
• About 3% of infants on nevirapine prophylaxis acquired HIV infection.
• All three cases were born to mothers who were diagnosed with HIV at delivery.
• Poor adherence to nevirapine and delayed HIV diagnosis were implicated.

## Introduction

Globally, by the end of 2022, there were about 39.0 million individuals living with HIV. The WHO African Region remained the hardest-hit, with an estimated 3.2% of adults living with HIV, representing over two-thirds of the global HIV-positive population [1]. Advanced HIV disease accounts for 3% of deaths in children below 5 years of age globally and 6% in sub-Saharan Africa. Despite scaling up of elimination of mother-to-child transmission (eMTCT) programs, the global rate of vertical HIV transmission after breastfeeding is still relatively high estimated at 8.6% [2] In Uganda, the final HIV vertical transmission rate including breastfeeding was estimated at 6.8% in 2021 from 12.1% in 2015 with early infant diagnosis (EID) at 74.5%. EID confers a substantial benefit to HIV-infected and HIV-uninfected infants and programs providing prevention of mother-to-child transmission (MTCT) but has been challenging to implement in resource-limited settings [3]. Over 90% of infection in children is acquired through MTCT [4].

The goal of prevention of MTCT (PMTCT) is to minimize new HIV infection to less than 2% with PMTCT interventions. In 2013, Uganda adopted Option B+ for PMTCT issued by the WHO, which provides for immediate initiation of antiretroviral therapy (ART) for pregnant women for life, regardless of cluster of differentiation 4 count, and mandates: (a) daily nevirapine prophylaxis for all HIV-exposed infants at birth, regardless of feeding modality, for 6 weeks; (b) EID through HIV DNA testing at 6 weeks of life, 9 months of life and 6 weeks after cessation of breastfeeding; and (c) rapid initiation of ART for all infants who test HIV positive. Additionally, all infants with an initial negative HIV DNA test result at 6 weeks of age are retested at 6 months of age. Despite the impressive benefits of the Option B+ strategy, implementation challenges, including cost burden and mother-baby pairs lost to follow-up threatened its overall effectiveness [4].

Nevirapine mono prophylaxis has proven efficacy, safety, and affordable cost making it the most preferred non-nucleoside reverse transcriptase inhibitor for pediatric prophylactic use in resource-limited settings [5]. The national PMTCT guidelines recommend daily nevirapine for the first 6 weeks of life starting as soon as possible after birth, preferably within 72 hours of birth. Nevirapine belongs to the family of non-nucleoside analog reverse transcriptase inhibitors. However, single substitutions at several reverse transcriptase positions have been reported to confer resistance to nevirapine [5]. Access to ART by pregnant women with HIV has significantly improved outcomes for exposed infants as evidenced by data from UNAIDS indicating that an estimated 100% of pregnant women with HIV in Uganda accessed ART in 2021 compared to data from 2016 where 97% accessed ART but only 38% of HIV-exposed infants received nevirapine for PMTCT [3].

Without ART, the risk of transmission of HIV during pregnancy and labor from mothers infected with HIV to their children is approximately 15-30%, additionally, prolonged breastfeeding is attributed to a 10-20% transmission risk. HIV disease progresses very rapidly in young children, often leading to death. Without care and treatment, one-third of infants die in 1^st^ year, and about half of children by the 2^nd^ year of life [3]. Advanced HIV disease was responsible for 17% of deaths among children in 2015 [4]. In 2021, about 4000 advanced HIV-related deaths were registered among children 0-14 years of age in Uganda [6]. Therefore, in this study, we aimed to determine the proportion of infants who tested positive for HIV despite receiving eMTCT services in a rural setting in Northern Uganda.

## Methods

### Study design and setting

We conducted a retrospective cohort study utilizing medical records from the EID register in the maternity unit of Lalogi Health Centre IV in Omoro district, Northern Uganda between January 2019 to June 2021.

### Study population

We included records of children born to HIV-infected mothers during pregnancy and delivery and followed each child for up to 18 months. Children born to HIV-positive mothers, who had been given nevirapine syrup prophylaxis and completed 18 months follow-up, were included in the study. Information was taken from the AIDS register of the hospital.

### Sample size

Lalogi had a total of 6630 children by 2021 June of which 180 were exposed to HIV perinatally. The EID register for the year 2019, 2020, and 2021 was considered for the study since 2021 entries alone was an insufficient sample size.

There were 165 HIV-exposed infants who were 18 months old by June 2021 and who had been given nevirapine. The sample was drawn from this cohort.

The sample size of 118 HIV-exposed children was calculated using the modified Kish and Leslie formula for finite population size, with an estimated proportion of HIV-exposed infants who used nevirapine being 91.6%, and type I error of 5%, and a SD at 95% CI (1.96).

### Sampling and data collection

A systematic random sampling was employed, with a sampling interval of (165/118.2) = 1.4, where every other one entry in the register was considered as they met the inclusion criterion since there is no 0.4 person. All the 118 sample was drawn from a frame of 165 in EID register entries of 2019 through 2021. Data was collected from the EID register using a questionnaire checklist designed in English. All entries were reviewed for eligibility.

### Data analysis

Data were entered into Microsoft 2016 and analyzed using the STATA version 16.0 analysis tool. Descriptive statistics were performed, and numerical data were summarized as mean and SD or median and interquartile range.

### Ethical considerations

Ethical approval and waiver of consent were obtained from Gulu University Research and Ethics Committee (approval number: GUREC -2022-381) and administrative clearance was obtained from Lalogi Health Center administration. The study was conducted in observance of the *Declaration of Helsinki.*

## Results

### Baseline characteristics

We enrolled 118 HIV-exposed children at birth and followed up until 18 months between January 2019 and June 2021. Of these, 54.2% (64) were female.

Overall, 113 (95.8%) participants had had their mothers diagnosed with HIV and initiated on ART before pregnancy. However, four (3.4%) mothers were diagnosed with HIV during delivery and one (0.8%) during antenatal care period, Table 1.

**Table 1:** Time of maternal HIV diagnosis.

Time of diagnosis of mother with HIV	Frequency	Percent (%)
Before pregnancy	113	95.8
During antenatal care	1	0.8
At delivery	4	3.4
**Total**	**118**	**100**

### Nevirapine prophylaxis

Of the 118 infant files reviewed, 110 (93.2%) were initiated on nevirapine prophylaxis on the day of birth, two (1.7%) were initiated after 2 weeks, and one (0.9%) each on the 4^th^ and 6^th^ weeks, Table 2. Overall, 111 (94.1%) infants were on nevirapine prophylaxis for 6 weeks, five (4.2%) for less than 6 weeks, and two (1.7%) for more than 6 weeks.

**Table 2:** Time-to-initiation of nevirapine prophylaxis.

Time, age	Gender		Total
	Female (%)	Male (%)	Total (%)
0-1 day	49 (41.5)	61 (51.7)	110(93.2)
7-10 days	2 (1.7)	2 (1.7)	4(3.4)
2 weeks	2 (1.7)	0 (0.0)	2(1.7)
4 weeks	0 (0.0)	1 (0.8)	1(0.8)
6 weeks	1 (0.8)	0 (0.0)	1(0.8)
**Total**	**54 (45.8)**	**64 (54.2)**	**118(100)**

### HIV polymerase chain reaction test outcomes

At first polymerase chain reaction (PCR) (6 weeks after birth), one (0.8%) participant was found to be HIV positive. At the second PCR (9 months after birth), two (1.7%) participants were found to be HIV positive, and at the third PCR (6 weeks after weaning off breastfeeding), none was HIV positive, Table 3. All the HIV RNA-positive children had a positive HIV rapid antibody test.

**Table 3:** HIV PCR results across breastfeeding choices of HIV-exposed infants.

Gender	Infant HIV results PCR test									N = 118
	Missing Results frequency			Negative frequency			Positive frequency			
	Complementary Feeding	Exclusive Breastfeeding	No longer Breastfeeding	Complementary Feeding	Exclusive Breastfeeding	No longer Breastfeeding	Exclusive Breastfeeding	Complementary Feeding	No longer Breastfeeding	
First PCR at 6 week										
Female	0	0	0	0	49	4	0	0	0	53
Male	0	0	0	2	60	2	1	0	0	65
**Total**	**0**	**0**	**0**	**2**	**109**	**6**	**1**	**0**	**0**	**118**
Second PCR at 9 months										
Female	1	0	0	48	0	0	0	1	0	50
Male	1	1	0	62	1	1	0	1	0	67
**Total**	**2**	**1**	**0**	**110**	**1**	**1**	**0**	**1**	**0**	**117**
Third PCR at 6 weeks after cessation of breastfeeding										
Female	0	0	2	11	0	36	0	0	0	49
Male	0	0	3	15	0	48	0	0	0	66
**Total**	**0**	**0**	**5**	**26**	**0**	**84**	**0**	**0**	**0**	**115**

PCR, polymerase chain reaction.

Of the three HIV breakthrough cases, one was male, and two were female. The mothers of all three children were diagnosed with HIV at delivery. Two children were on nevirapine prophylaxis for <6 weeks and one for up to 12 weeks. Overall, 115 (97.5%) participants had exclusive breastfeeding, two (1.7%) had complementary feeding, and one (0.8%) was no longer breastfeeding.

### Overall 18-month clinical outcomes

Of the 118 infants, 109 (92.4%) were negative, five (4.2%) were lost to follow-up, three were confirmed HIV positive and referred for ART, and one (0.8%) died negative due to malaria, Table 4.

**Table 4:** Infants’ HIV status at 18 months of age.

Infants’ HIV status at 18 months	Frequency	Percent
Discharge negative	109	92.4
Confirmed HIV+ and referred for antiretroviral therapy	3	2.5
Lost to follow-up	5	4.2
Died positive	0	0.0
Died negative	1	0.8
**Total**	**118**	**100**

## Discussion

In this study, we aimed to determine the proportion of infants who tested positive for HIV despite receiving eMTCT services in a rural setting in Northern Uganda. The study is hinged on review of EID register in the maternity unit of Lalogi Health Centre IV, which focused on the HIV breakthrough infection in infants by 18 months of age who were on nevirapine syrup prophylaxis for 6 months as recommended by WHO and Uganda Consolidated HIV Guideline of 2020, however, it is of note that Uganda uses monotherapy of nevirapine prophylaxis.

The HIV breakthrough infection prevalence was at 2.5% in HIV-exposed children at birth in the Omoro district which is lower than the reported 5.8% of children who got infected due to MTCT in the year 2020 by the Ministry of Health [5]. This could be attributed to the 4.2% of the children who had poor adherence to nevirapine syrup prophylaxis and high-risk HIV exposure that is, diagnosis of mother at delivery and initiation of ART during breastfeeding. Poor adherence and late ART initiation result from several factors such as inadequate information on eMTCT of HIV, failure to attend antenatal care, and mixed feeding of HIV-exposed infants. As such, the factors contribute to the 5500 reported new MTCT of HIV in Uganda by UNAIDS in 2021 [6].

Our results are comparable to findings from studies conducted in other countries where prevalence of HIV infection among HIV-exposed infants was estimated at 3.8% and 3.6% in Ethiopia [7] and Swaziland [8], respectively. Whereas relatively higher prevalence is estimated at 7.7% and 7.8% in Ethiopia and India, respectively. The observed differences could be due to the differences in the sample sizes and data collection methods. In our study, the reviewed files reflected a good reported adherence to nevirapine syrup for the first 6 weeks. However, 4.2% of the children had poor reported adherence to nevirapine syrup prophylaxis coupled with complementary feeding in the 9^th^ month of age and, five (4.2%) of the children's mothers were initiated on ART post-delivery. A combination of these factors increases the risk of children acquiring HIV from their mothers which is consistent with a Ministry of Health report indicating that 20% of new pediatric infections as of 2021 were because of delayed ART initiation among mothers living with HIV [9]. However, timely intervention is an effective preventive method, for instance, about 21,000 new HIV infections were averted due to PMTCT in Uganda.

There was a 100% utility of dry blood spot for the diagnosis of the children, this is consistent with a finding by Paranjpe et al. [3] in their study on EID Of HIV which showed 100% concordance between dry blood spot and whole blood PCR, and by the cumulative first and second DNA PCR test, three (2.5%) of the infants had acquired HIV from their mothers all of which were high risk exposed infants.

Only one HIV infection was registered at the first PCR test possibly due to antenatal or birth infection and good use of nevirapine prophylaxis. However, by the 9^th^ month, poorly reported adherence in 1.7% of the children could have contributed to the increase of prevalence from 0.8-2.5%. This implies in 118 HIV-exposed infants, there is a 2.5% prevalence rate of breakthrough HIV infection, which contributes positively to the reduction of under 5 years old to as low as 25 per 1000 life birth as by the Sustainable Development Goal of the United Nations by the year 2030 [10].

This study was not without limitations. Missing data from the registers, loss to follow-up, and only infants who had delivered at the health center and who had received nevirapine were included so vulnerable infants from home deliveries and who did not get PMTCT services were missed, could have caused loss of important data on HIV breakthrough infection affecting the quality of the study findings. Purposive sampling of all the patients in the EID register may have posed a bias of representation of patients in terms of origin or home location however, we used the register information as a random distribution of the respondent to select the respondents from the EID register to minimize the biases.

## Conclusion

In this study, we found that while eMTCT services were largely successful in minimizing vertical transmission of HIV in the rural setting in Northern Uganda, there were still some cases of breakthrough HIV infection associated with non-adherence to nevirapine prophylaxis and delayed HIV diagnosis. Therefore, adhering to the national guidelines on nevirapine prophylaxis for at least 6 weeks for children born to mothers with HIV is recommended to further reduce the risk of vertical transmission of HIV.

## Declarations of competing interest

The authors have no competing interests to declare.
